# A Carcinoma of Unknown Primary (CUP) Patient Treated Successfully With Immunotherapy Upon Recognition of Deficient Mismatch Repair Signature on Liquid Biopsy: A Case Report

**DOI:** 10.7759/cureus.27184

**Published:** 2022-07-23

**Authors:** Ronald E Cox, Sakti Chakrabarti

**Affiliations:** 1 Oncology, Medical College of Wisconsin, Milwaukee, USA; 2 Medical Oncology (GI Oncology), Medical College of Wisconsin, Milwaukee, USA

**Keywords:** circulating tumor dna (ctdna), liquid biopsy, immune checkpoint inhibitor, immunotherapy, carcinoma of unknown primary (cup)

## Abstract

Carcinoma of unknown primary (CUP) refers to a heterogeneous group of metastatic tumors in which the origin of the tumor is undetermined. Patients with CUP have limited treatment options and an overall poor prognosis. Genomic profiling of the tumor tissue sometimes reveals actionable mutations leading to targeted treatment opportunities. However, sufficient tumor tissue is not often available for genomic profiling. In such situations, circulating tumor DNA (ctDNA)-based tumor genomic profiling may be valuable, although robust data do not exist to support the role of ctDNA testing in patients with CUP. Herein, we report a patient with CUP who was successfully treated with an immune checkpoint inhibitor (ICI) after ctDNA testing revealed a deficient mismatch repair (dMMR) tumor.

## Introduction

Carcinoma of unknown primary (CUP) is a heterogeneous group of metastatic cancer in which the origin of the tumor is unknown despite extensive evaluations, including imaging studies, serum biomarker studies, endoscopies, and tumor evaluation with immunohistochemistry [1]. CUP constitutes 3-5% of worldwide cancer diagnoses with decreasing incidence since the early 1990s, likely reflecting improvements in diagnostic modalities that help detect the primary cancer site [1]. The treatment of CUP primarily consists of platinum-based combination chemotherapies that achieve a response rate of 20% to 40% and a median survival of 6 to 8 months [2]. As the prognosis of patients with CUP treated with conventional chemotherapy is poor, interest has been emerging in exploring novel targeted therapies and immunotherapies for this group of patients. Genomic profiling of the tumor tissue by next-generation sequencing (NGS) can present targeted therapy opportunities. Circulating tumor DNA (ctDNA)-based tumor genomic profiling (liquid biopsy) could be an alternative option if sufficient tumor tissue is not available for tissue NGS [3]. We present a case report in which a deficient mismatch repair (dMMR) status/microsatellite instability-high (MSI-H) signature was detected utilizing a liquid biopsy test that led to successful treatment with an immune checkpoint inhibitor (ICI).

## Case presentation

**Figure 1:**
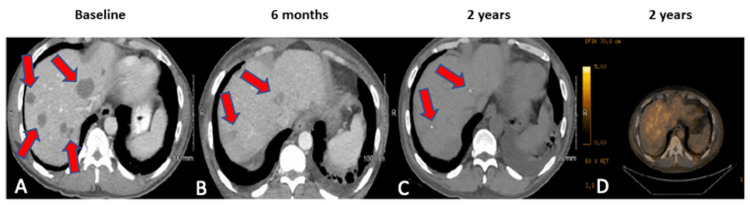
Serial CT scans The serial scans show progressive tumor shrinkage with eventual complete remission. (A) CT performed at diagnosis. (B) CT performed 6 months after the initiation of immunotherapeutic treatment. (C) Two-year follow-up CT scan performed without contrast as the patient developed nephropathy. (D) PET-CT-confirmed continued complete response at the 2-year mark.

A 46-year-old African American man with a diagnosis of CUP presented for a second opinion. He initially presented to an oncologist with progressively worsening abdominal pain, nausea, loss of appetite, and weight loss. He also had long-standing poorly controlled type II diabetes mellitus, hypertension, chronic renal failure with slightly elevated creatinine, and peripheral neuropathy likely resulting from the diabetes mellitus. The examination was remarkable for significant tenderness in the epigastric and the right upper quadrant of the abdomen. His overall activity level was significantly limited, with an Eastern Cooperative Oncology Group (ECOG) performance status of 2. He underwent an extensive workup that consisted of a complete blood count (CBC), comprehensive metabolic panel (CMP), serum tumor markers, and contrast-enhanced computed tomography (CT) scan of the chest, abdomen, and pelvis. CBC was remarkable for moderate anemia, and CMP was remarkable for a slightly elevated alkaline phosphatase with normal total bilirubin and transaminase levels. CT scan showed multiple (more than 20) low-density liver lesions measuring from less than a centimeter (cm) to several centimeters in size, with the largest measuring nearly 4 cm (Figure 1).

The carcinoembryonic antigen (CEA) level was also elevated at 469 ng/ml, the normal range being < 5 ng/ml. The patient underwent a percutaneous ultrasound-guided core needle biopsy of one of the liver lesions, which showed adenocarcinoma consistent with a colonic primary. However, a colonoscopy performed right after the liver biopsy did not show any primary colonic tumor. Esophagogastroduodenoscopy was unremarkable as well. A positron emission tomography (PET)/CT scan from skull base to mid-thigh showed multiple fluorodeoxyglucose (FDG)-avid hepatic metastases and lymphadenopathy (retrocrual and left retroperitoneal lymph nodes). There was no family history suggestive of Lynch syndrome. He was treated with four cycles of modified FOLFOX (5-fluorouracil, leucovorin, and oxaliplatin) and bevacizumab. A CT scan after four cycles of chemotherapy showed disease progression. At this point, the patient presented to us for a second opinion.

We ordered genomic profiling of the tumor, but the tissue was insufficient for NGS and inadequate for immunostaining to evaluate MMR status. Consequently, a liquid biopsy was obtained utilizing the commercially available Guardant360 CDx test (Guardant Health, Inc., Redwood City, California, USA). The liquid biopsy revealed a microsatellite instability-high (MSI-H) tumor. Treatment with a programmed death 1 (PD1) blocker pembrolizumab (200 mg intravenously every 21 days) was initiated at this time. A CT scan after the third cycle showed stable disease. He received an additional three cycles of pembrolizumab, and a CT scan after cycle 6 showed a remarkable shrinkage of the hepatic lesions and the lymph nodes. He continued treatment for an additional seven cycles, leading to progressive shrinkage of liver lesions to eventual complete remission (CR) (Figure 1). However, after completing cycle 13 of pembrolizumab over approximately 10 months, his creatinine began to rise from baseline. Pembrolizumab was held, and a referral was made to nephrology. Nephrology workup, which included a renal biopsy, revealed acute interstitial nephritis (AIN), likely secondary to immunotherapy, and signs of chronic diabetic kidney disease. He was taken off pembrolizumab and followed closely with serial scans. His kidney function stabilized, and subsequent scans, including a PET-CT scan, showed CR. Currently, he is being followed without any active anti-cancer therapy, with the scans showing continued CR approximately 30 months after the initiation of pembrolizumab.

## Discussion

We describe a patient with CUP who did not respond to systemic chemotherapy and subsequently underwent ctDNA-based genomic profiling that revealed an MSI-H tumor. The recognition of MSI-H signature in the tumor based on the liquid biopsy report led to treatment with an ICI, pembrolizumab, that resulted in complete resolution of the disease. Despite discontinuing the treatment with pembrolizumab upon a diagnosis of ICI-induced nephropathy, an Immune-Related Adverse Event (irAE), patients showed a durable response.

This case report highlights that an attempt to employ novel targeted therapy and immunotherapy should be made in each patient with a diagnosis of CUP, as CUP treated with conventional chemotherapy portends a poor prognosis [2,4]. Lombardo et al. performed a large-scale systematic review to investigate the utility of NGS in detecting actionable driver mutations in CUP patients [5]. The systematic review included 3,161 CUP patients, and 47.3% of patients harbored a potentially targetable alteration. Furthermore, if tissue-based genomic profiling is not feasible because of the insufficient amount of tumor tissue in the biopsy specimen, ctDNA-based genomic profiling or “liquid biopsy” should be attempted. A large retrospective study based on liquid biopsy in CUP patients reported the presence of a potentially targetable alteration in 99.7% (289/290) of patients [3].

A plethora of studies has reported robust anti-tumor activity of ICIs in MSI-H/dMMR tumors, both in treatment-naive and previously treated patients, irrespective of the site of tumor origin [6,7]. Consequently, a search for a predictive biomarker for immunotherapy response should be done in any solid tumor patients, including CUP patients. As illustrated in the current case report, identifying MSI-H signature could open up gratifying treatment opportunities. It is also important to emphasize that detection of MSI-H signature based on liquid biopsy is feasible, and MSI-H signature detected by liquid biopsy predicts robust and durable anti-tumor response to ICIs [8,9].

Another important aspect of immunotherapy in MSI-H solid tumors is that the responses are durable even after immunotherapy is discontinued, as seen in the current patient [7]. It is reasonable to withhold immunotherapy in the event of irAEs if the tumor shows no progression. As our patients showed continued tumor control on serials scans, we have not resumed treatment.

## Conclusions

This case report highlights that ICIs can be successfully utilized in the treatment of patients with CUP harboring MSI-H signature. Tumor genomic profiling, either by tissue NGS or liquid biopsy, should be an integral part of the CUP workup. ctDNA-based genomic profiling may reveal actionable targets leading to novel treatment opportunities and improved outcomes.
